# Identification of dysregulation of atrial proteins in rats with chronic obstructive apnea using two‐dimensional polyacrylamide gel electrophoresis and mass spectrometry

**DOI:** 10.1111/jcmm.14131

**Published:** 2019-02-12

**Authors:** Jacob C. Lux, Devika Channaveerappa, Roshanak Aslebagh, Timothy A. Heintz, Meredith McLerie, Brian K. Panama, Costel C. Darie

**Affiliations:** ^1^ Department of Experimental Cardiology Masonic Medical Research Laboratory Utica New York; ^2^ Biochemistry and Proteomics Group, Department of Chemistry and Biomolecular Science Clarkson University Potsdam New York

**Keywords:** animal proteomics, apnea, cardiovascular system, hypoxia, metabolism, rat, two‐dimensional electrophoresis

## Abstract

Obstructive sleep apnea (OSA) affects an estimated 20% of adults worldwide and has been associated with electrical and structural abnormalities of the atria, although the molecular mechanisms are not well understood. Here, we used two‐dimensional polyacrylamide gel electrophoresis (2D PAGE) coupled with nanoliquid chromatography‐tandem mass spectrometry (nanoLC‐MS/MS) to investigate the proteins that are dysregulated in the atria from severe and moderate apnea when compared to control. We found enzymes involved in the glycolysis, beta‐oxidation, electron transport chain and Krebs cycle to be down‐regulated. The data suggested that the dysregulated proteins may play a role in atrial pathology developing via chronic obstructive apnea and hypoxia. Our results are consistent with our previous 1D‐PAGE and nanoLC‐MS/MS study (Channaveerappa et al, J Cell Mol Med. 2017), where we found that some aerobic and anaerobic glycolytic and Krebs cycle enzymes were down‐regulated, suggesting that apnea may be a result of paucity of oxygen and production of ATP and reducing equivalents (NADH). The 2D‐PAGE study not only complements our current study, but also advances our understanding of the OSA. The complete mass spectrometry data are available via ProteomeXchange with identifier PXD011181.

## 1 INTRODUCTION

Obstructive sleep apnea (OSA) is characterized by repeated cessation in respiration (apnea) in the upper airway during sleep lasting at least 10 seconds. Apneas occur as a result of temporary pharyngeal collapse or narrowing, resulting in decreased blood oxygen saturation.^1^ OSA has been associated with headache, daytime sleepiness, obesity, depression, arthritis, type 2 diabetes mellitus, arteriosclerosis, atherosclerosis, hypertension, atrial arrhythmia and sudden cardiac death.^1^, ^2^ Although OSA has been closely associated with atrial arrhythmia, atrial enlargement, increased P wave duration and decreased atrial voltage; little is known about the molecular pathways causing these pathologies.^3^, ^4^ Many animal models do not address both the obstructive and hypoxemic components of OSA in conscious, free roaming animals. In order to accurately reproduce the effects of OSA as observed in the clinic, we have employed a surgical model involving a silicone obstructive device implanted in the trachea of conscious, free roaming rats.^3^

Here, we used 2D PAGE coupled with nanoLC‐MS/MS as a complementary approach to investigate the proteins that are dysregulated in the atria from severe and moderate apnea when compared to control. While the 1D‐PAGE approach compared the whole atrial proteomes from the severe OSA, moderate OSA and controls,^3^ the 2D‐PAGE approach allowed us to identify only the dysregulated proteins from these conditions. Furthermore, 2D‐PAGE also allowed us to identify different protein isoforms, already demonstrated in others’ work.^5^ In this study, we not only found that the entire glycolytic pathway and Krebs cycle are down‐regulated, but also found evidence that additional enzymes involved in the beta‐oxidation, electron transport chain and Krebs cycle anaplerotic reactions were also down‐regulated. Other protein dysregulations identified are involved in metabolic, structural or inflammatory pathways, suggesting that these proteins may play a role in atrial pathology developing via chronic obstructive apnea and hypoxia.

## 2 MATERIALS AND METHODS

The rats and the rat model setup (Figure S1) and the sample preparation method used in this study was described previously.^3^, ^6^ Atrial homogenates of control (n = 2), moderate (n = 2) and severe (n = 2) apnea were analysed by 2D PAGE and nanoLC‐MS/MS analysis. A total of 2293 protein spots were compared for statistical differences, of which 208 spots having a fold increase or decrease of ≥1.7 and a *P* ≤ 0.05, or a fold increase or decrease of ≥3.0 were selected for nanoLC‐MS/MS analysis, as previously described.^6^ Raw data were processed as in,^6^ using NCBInr rat (*rattus*) database. The mass spectrometry proteomics data have been deposited to the ProteomeXchange Consortium via PRIDE partner repository with dataset identifier PXD011181 and 10.6019/PXD011181.^7^

## 3 RESULTS

**Figure 1:**
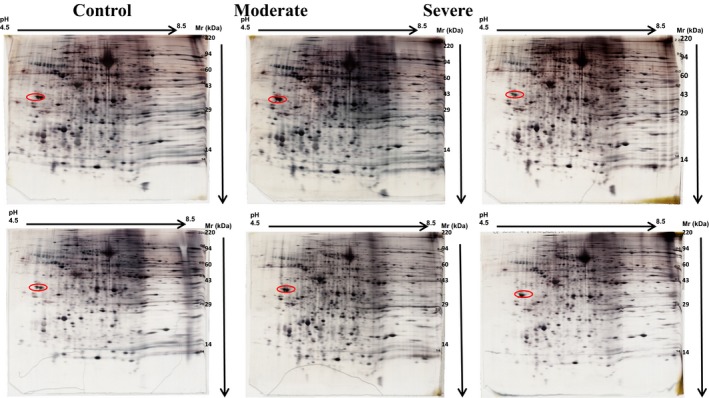
Images of control, moderate, and severe apnea silver stained 2D polyacrylamide gels. The circles on the left side of each 2D polyacrylamide gel indicate the location of the IEF internal standard, tropomyosin with a Mr of 33 000 and pI of 5.2

Comparison of the protein pattern between severe OSA versus control and moderate OSA versus control was done using 2 biological replicates for each condition. Two Coomassie and 1 silver stained gels were run for each of the 6 samples (2 controls, 2 moderates and 2 severe samples). Figure S2 shows an example of one Coomassie stained gel for each sample and Figure 1 shows the silver stained 2D PAGE gel images of the 3 conditions in duplicates. Figure S3 depicts the 2D gel difference image of averaged severe apnea vs averaged control where the spots increased in severe are shown in blue and the spots decreased in severe are shown in red. A summary and comparison of all the proteins identified from the spots of severe, moderate and control gels are shown in Tables [Link], [Link], [Link]. A few important proteins dysregulated between severe apnea, moderate apnea and control conditions, their function and their possible association with OSA disease are discussed below.

### 3.1 Dysregulated proteins in severe apnea samples, compared to controls

Up‐regulated proteins in severe apnea samples when compared to controls are shown in Figure S4A. Some examples of the dysregulated proteins include creatine kinase (CK) M‐type (9.8‐fold), aldose reductase (AR) (9.8‐fold), long‐chain specific acyl‐CoA dehydrogenase, mitochondrial precursor (9.8‐fold), alpha‐actinin‐2 (6.7‐fold), alpha‐myosin heavy chain, partial (6.2‐fold), myomesin‐1 (skelemin) (4.8‐fold), myosin light chain 4 (4.7‐fold), myomesin‐2 (4.6‐fold); myosin light chain (4.0‐fold) and heat shock protein 70 (3.9‐fold).

Down‐regulated proteins in severe apnea samples when compared to control samples are shown in Figure S4B and include cytochrome c oxidase (CCO) subunit 4 isoform 1, mitochondrial precursor (−9.6‐fold), troponin I, cardiac muscle (−3.3‐fold), triosephosphate isomerase (−3.4‐fold), ADP/ATP translocase (AAC) 1 (−3.4‐fold); Ba1‐647 (−3.4‐fold), L‐lactate dehydrogenase B chain (−3.4‐fold), malate dehydrogenase, cytoplasmic (−3.4‐fold), and long‐chain specific acyl‐CoA dehydrogenase, mitochondrial precursor (−3.0‐fold).

### 3.2 Dysregulated proteins in moderate apnea samples, compared to controls

Up‐regulated proteins in moderate apnea samples when compared to controls are shown in Figure S5A. Some of these proteins include Annexin V (6.3‐fold), isoform CRA_a (5.1‐fold), ATP synthase, mitochondrial F1 complex, alpha subunit (6.3‐fold); ATP synthase beta subunit (4.4‐fold), cytochrome b‐c1 complex subunit 1, mitochondrial precursor (3.8‐fold), pyruvate dehydrogenase E1 component subunit beta, mitochondrial precursor (3.1‐fold) and aspartate aminotransferase, mitochondrial precursor (2.3‐fold).

Down‐regulated proteins in moderate apnea samples when compared to controls are shown in Figure S5B and include HSP 90‐beta (−12.5‐fold); long‐chain specific acyl‐CoA dehydrogenase, mitochondrial precursor (−6.9‐fold), transcriptional adapter 2‐alpha (−6.9‐fold); alpha actinin 2 (−7.6‐fold), cytochrome b‐c1 complex subunit 2, mitochondrial precursor (−5.5‐fold), vinculin, isoform CRA_a (−3.7‐fold), NDRG1 related protein NDRG2a2 (−3.6‐fold) and myristoylated alanine‐rich C‐kinase substrate (MARCKS) (−4.0‐fold).

Additionally, many enzymes associated with aerobic metabolic pathways like glycolysis, Krebs cycle and anaerobic respiration were dysregulated in severe and moderate apnea when compared to control which was also identified to be dysregulated in our 1‐D analysis.^3^

### 3.3 Dysregulated proteins in severe apnea compared to moderate apnea

Up‐regulated proteins in severe apnea samples when compared to moderate apneas are shown in Figure S6 and include gelsolin precursor (10.6‐fold), lon protease homolog (IONP1), mitochondrial precursor (10.6‐fold), E3 ubiquitin‐protein ligase RNF181 (10.6‐fold), Myomesin‐1 (skelemin) (4.8‐fold) and Myomesin‐2 (4.8‐fold). No significant down‐regulation was observed in severe apnea samples when compared to moderate apnea samples.

### 3.4 Protein charge heterogeneity on 2D PAGE

Proteins expressed from a single gene can undergo different post‐translational modifications (PTMs),^8^ such as acetylation, methylation, phosphorylation or nitrosylation. These modifications alter the pI of the proteins with a slight change in the molecular weight which will induce a spot shift in the 2D gels across the pH gradient.^9^ Hence, the protein products can migrate to multiple spots on the gel. This can be observed in our study where we have found the same protein to be both up‐regulated and down‐regulated in severe and/or moderate when compared to control. For example, protein AR was found to be up‐regulated in severe when compared to control in spot no. 1516 (Table S1) as well as down‐regulated in spot no. 1485 and 1489 (Table S1). The different spots are observed for the same protein because of its charge heterogeneity caused by the PTMs, suggesting that OSA is not only a dysregulation of the levels of proteins, but also the proteins’ PTMs.

## 4 DISCUSSION

Apnea has also been found to cause myocardial dysfunction.^10^ We used 2D‐PAGE and LC‐MS/MS analysis to further define proteomic dysregulation as it occurs in this model. We have discovered a number of mitochondrial precursors and proteins to be down‐regulated in severe and moderate apnea samples when compared to controls, indicating possible mitochondrial dysfunction. This correlates with studies on heart failure energetics, also indicating decreased mitochondrial efficacy and mitochondrial proteomic dysregulation.^11^, ^12^ Increased regulation of structural proteins has been observed, such as tubulin and alpha myosin, as well as proteins that are involved in apoptotic mechanisms. Proteins involved in lipid metabolism, such as Long chain acetyl CoA dehydrogenase, have increased, contrary to current literature on fatty acid metabolism in the diseased heart.^12^, ^13^ Increased regulation of heat shock proteins were observed, indicating possible compensatory activity, although antioxidants such as peroxiredoxin 3 have been down‐regulated. Multiple proteins known for pro‐arrhythmic interactions appear dysregulated including structural, GPCR, and sarcomeric proteins.^4^, ^14^, ^15^ Although many of the proteomic changes observed are indicative of cardiomyopathy and may enhance arrhythmias, this model does not match the hypoxic cardiomyopathy phenotype entirely, possibly because of additional factors related to airway obstruction and global hypoxia.^12^, ^16^

While in our previous study that employed 1D‐PAGE followed by nanoLC‐MS/MS analysis, we focused on a comparison of the full proteomes of the atrial tissues from control, moderate apnea and severe apnea, in current study that employed 2D‐PAGE followed by nanoLC‐MS/MS analysis, we focused only on the proteins that are dysregulated in the atrial tissues from control, moderate apnea and severe apnea. Therefore, the two studies could not only confirm, but also complement each other and produced also a more accurate representation of the proteomic changes as they occur in the atria. This is also evidenced by the large number of dysregulated identified proteins in 2D‐PAGE, which greatly complements our previous study.^3^ Specifically, in the 1D‐PAGE study we found that some of the enzymes involved in the aerobic and anaerobic glycolysis, as well as in the Krebs cycle are down‐regulated.

**Figure 2:**
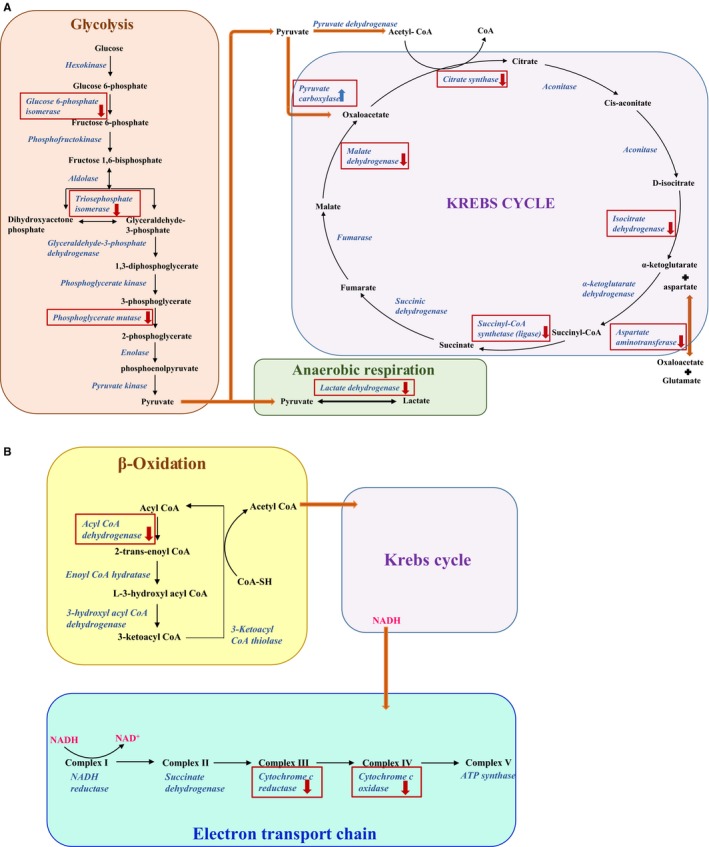
A, Outline of the aerobic (Glycolysis and Krebs cycle) and anaerobic (lactic acid fermentation) respiratory pathways depicting the down‐regulation of enzymes involved (shown in red downward arrow) in severe and moderate apnea when compared to control. The down‐regulated enzymes with an asterisk represents the findings from the 1‐D proteomic results, enzymes with two asterisks represent the findings from the current 2‐D proteomic results and the enzymes with a plus sign are found to be down‐regulated in both 1‐D and 2‐D proteomic analysis. B, Depiction of the β‐oxidation pathway and electron transport chain showing all the enzymes down‐regulated (shown in red downward arrow) in severe and moderate apnea condition when compared to control. The down‐regulated enzymes with an asterisk represents the findings from the 1‐D proteomic results, enzymes with two asterisks represent the findings from the current 2‐D proteomic results and the enzymes with a plus sign are found to be down‐regulated in both 1‐D and 2‐D proteomic analysis

In the current 2D‐PAGE study, additional enzymes involved in the aerobic glycolysis (Krebs cycle, electron transport chain), anaplerotic reactions (enzymes involved in the replenishment of the metabolites, ie oxaloacetate, alpha‐keto‐glutarate, succinylCoA) involved in the Krebs cycle and beta‐oxidation were also found, suggesting that the OSA‐induced oxygen deprivation of the heart induced down‐regulation of the entire glycolytic pathway, Krebs cycle, anaplerotic reactions, electron transport chain and beta‐oxidation of the fatty acids. For example, citrate synthase, which plays an important role as the first enzyme in the citric acid cycle by converting oxaloacetate and acetyl CoA to citrate and CoA‐SH^17^ is down‐regulated in various types of heart failure.^12^ In addition, mitochondrial dysfunction and citrate synthase down‐regulation have been shown to be exacerbated by atrial fibrillation.^11^ In our study, not only citrate synthase, but also the entire glycolytic pathway were down‐regulated. A comparative summary of the dysregulated enzymes in these pathways in our 1D‐PAGE^3^ and our current 2D‐PAGE experiments is shown in Figure 2.

Overall, we believe our model accurately recreates the cardiac effects of OSA as it occurs in humans. This model also led to identification of protein dysregulations that may indicate a potentiation for atrial arrhythmia, decreased atrial compliance, increased inflammation and risk of cardiomyopathy. This pilot study and methodology will be used as a baseline criterion to compare the protein dysregulations in severe apnea and moderate apnea with control using many biological and technical replicates.

## CONFLICT OF INTEREST

The authors declare no conflicts of interest related to this work.
