# Supplementary figures and images for: Identification of dysregulation of atrial proteins in rats with chronic obstructive apnea using two‐dimensional polyacrylamide gel electrophoresis and mass spectrometry
Source: J Cell Mol Med. 2019 Feb 12;23(4):3016–20. doi: 10.1111/jcmm.14131 (PMC6433690; doi:10.1111/jcmm.14131)

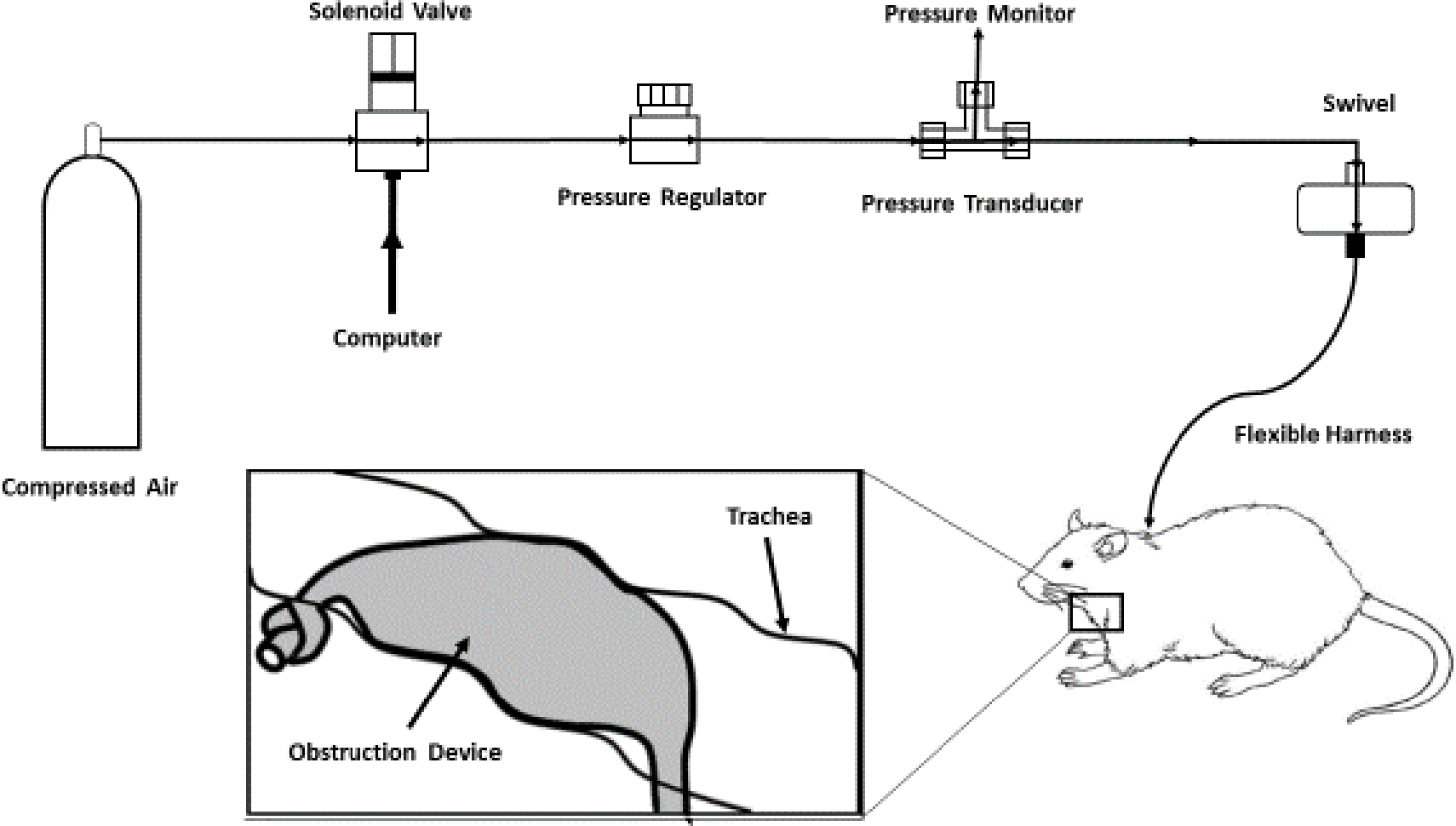

Supplement: Supplementary file 1 [file JCMM-23-3016-s001.tif]

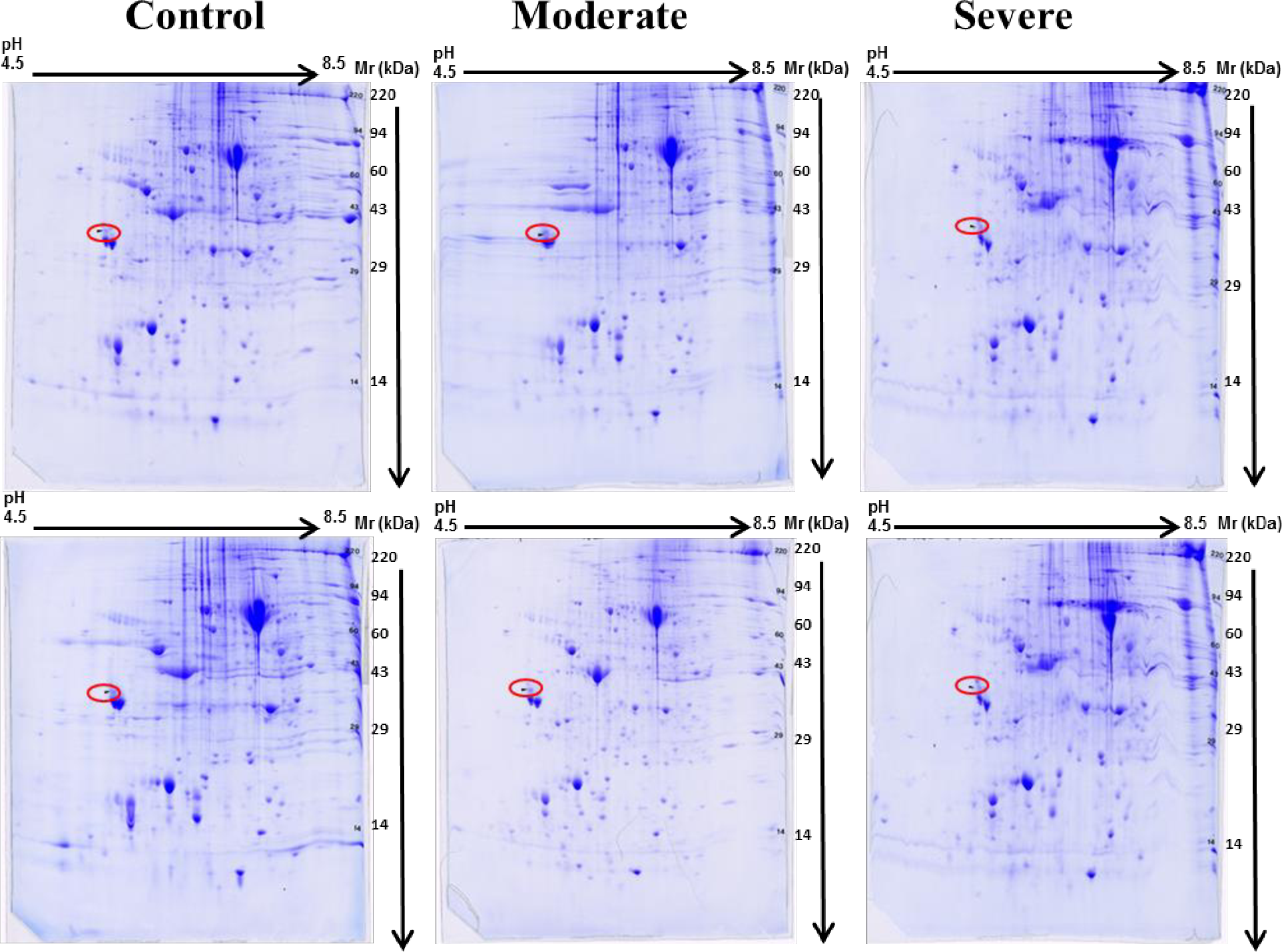

Supplement: Supplementary file 2 [file JCMM-23-3016-s002.tif]

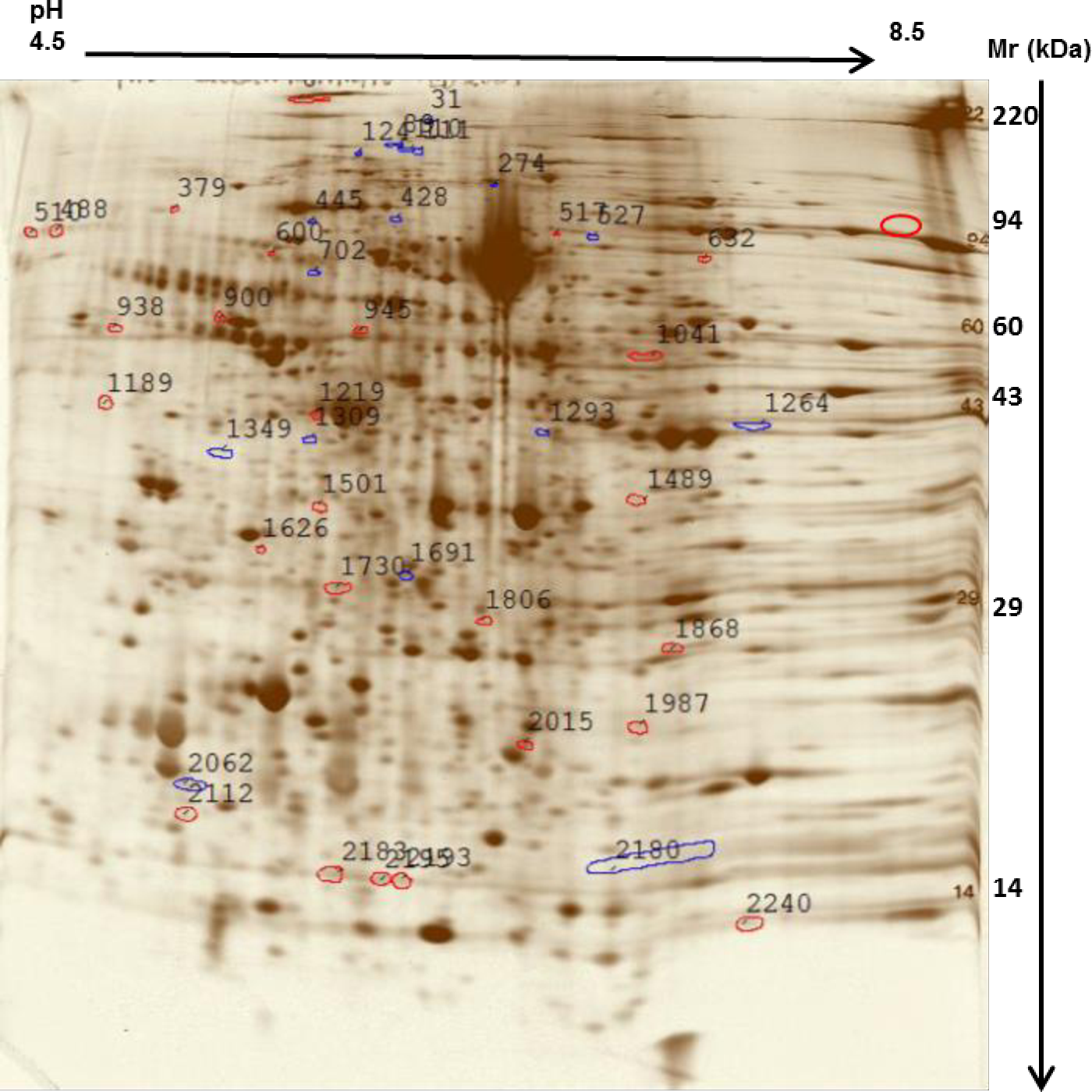

Supplement: Supplementary file 3 [file JCMM-23-3016-s003.tif]

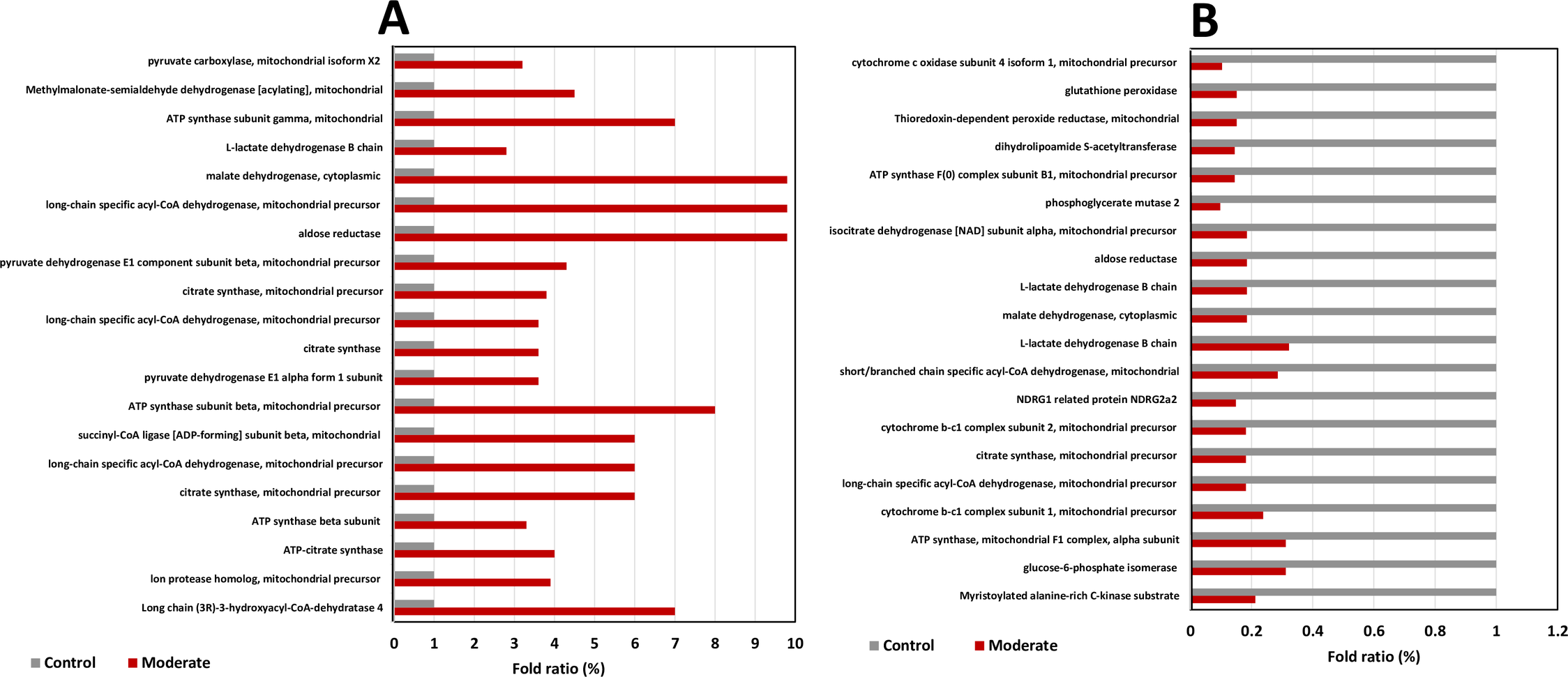

Supplement: Supplementary file 4 [file JCMM-23-3016-s004.tif]

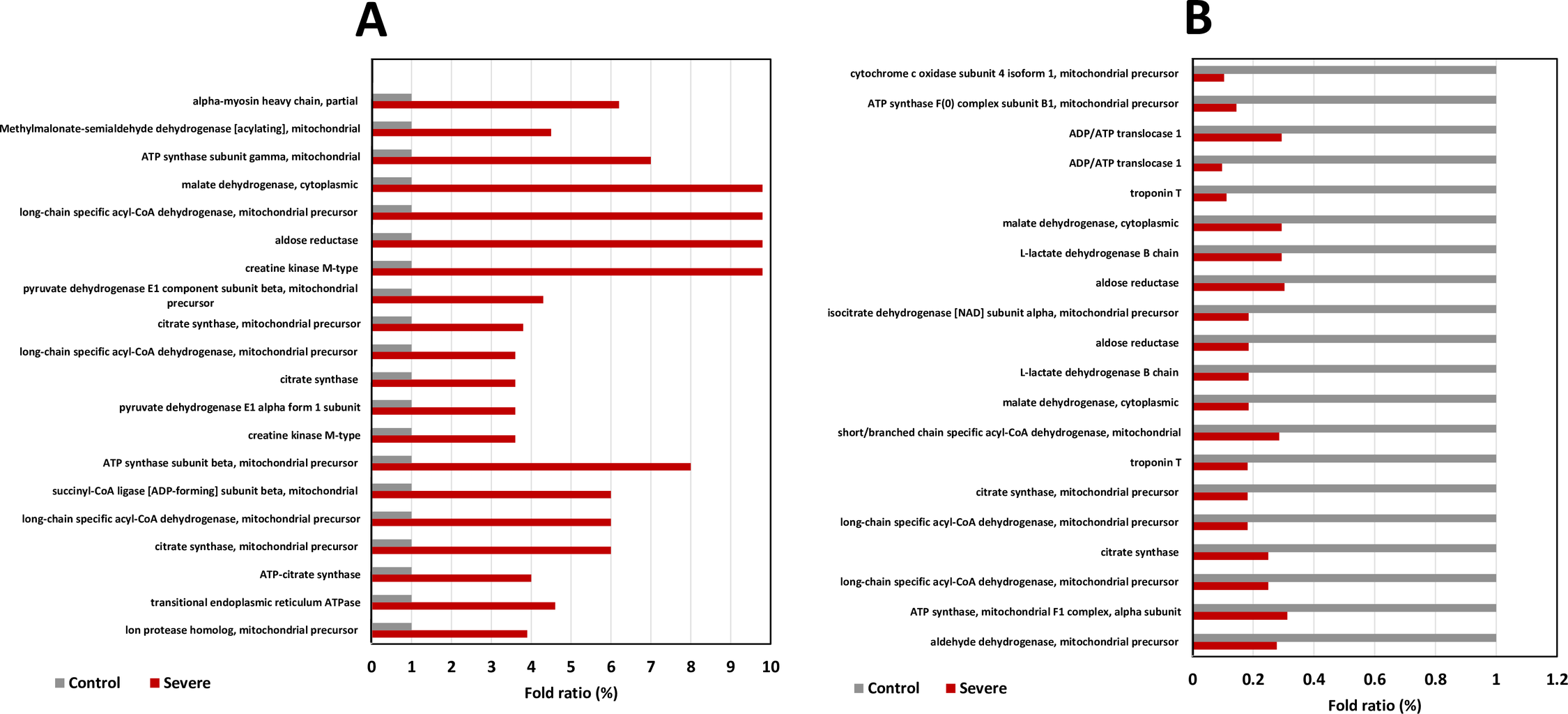

Supplement: Supplementary file 5 [file JCMM-23-3016-s005.tif]

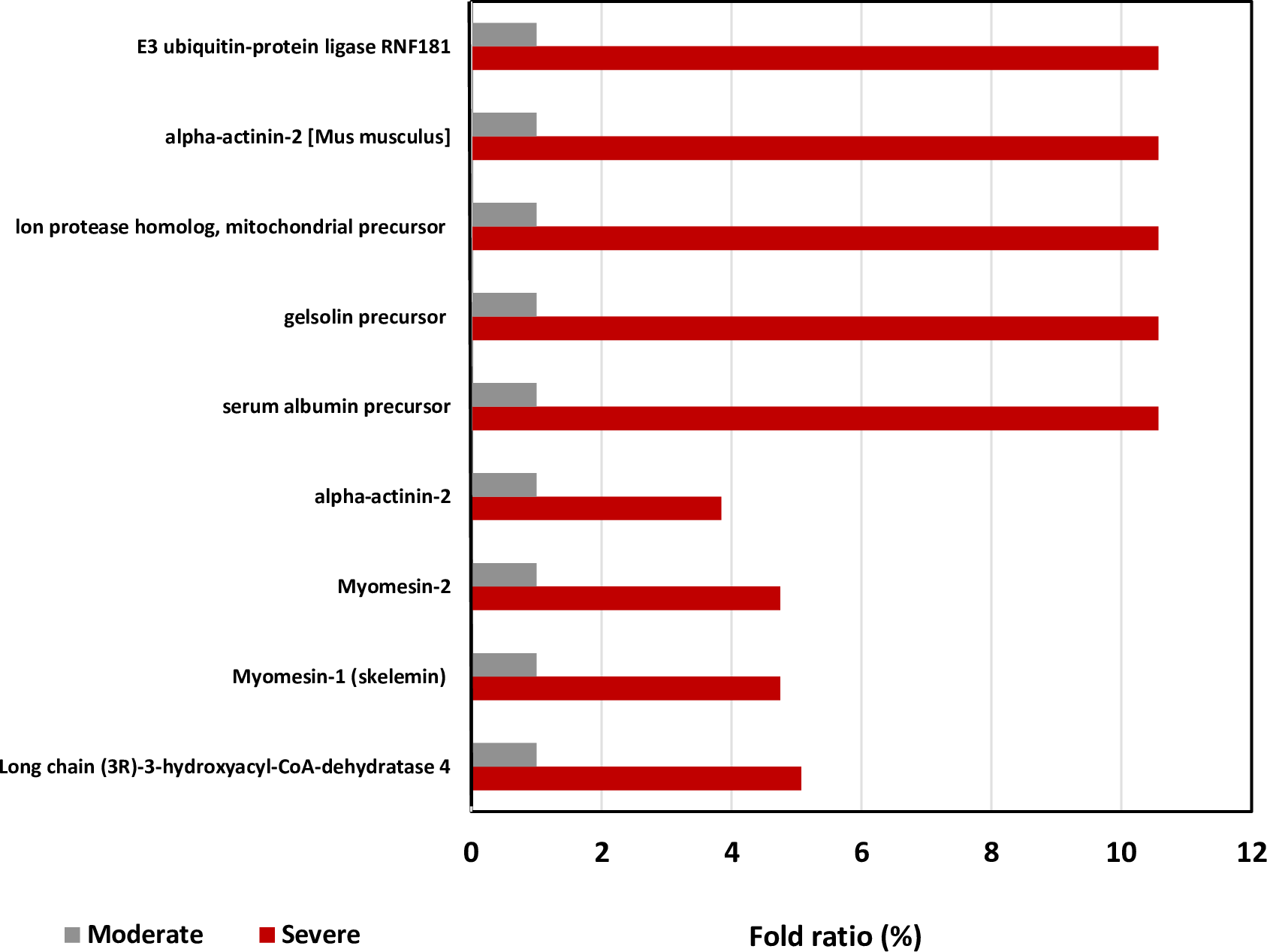

Supplement: Supplementary file 6 [file JCMM-23-3016-s006.tif]
