# Supplementary material for: Identification of dysregulation of atrial proteins in rats with chronic obstructive apnea using two‐dimensional polyacrylamide gel electrophoresis and mass spectrometry
Source: J Cell Mol Med. 2019 Feb 12;23(4):3016–20. doi: 10.1111/jcmm.14131 (PMC6433690; doi:10.1111/jcmm.14131)
Supplement: Supplementary file 8 [file JCMM-23-3016-s008.docx]

| **Spot #** | **Protein** | **NCBInr Accession no** | **Mr (kDa)** | **Fold Change** | **P value** |
| --- | --- | --- | --- | --- | --- |
| 10 | Long chain (3R)-3-hydroxyacyl-CoA-dehydratase 4 | gi\|157822059 | 27667 | 7.0 | 0.387 |
| 31 | Filamin-C | gi\|300797978 | 294072 | 4.1 | 0.033 |
| 46 | Myomesin-2 | gi\|281306803 | 166527 | 3.4 | 0.090 |
| 46 | Myomesin-1 (skelemin) | gi\|149036289 | 150969 | 3.4 | 0.090 |
| 46 | Neuroblast differentiation-associated protein (AHNAK) | gi\|77628165 | 21245 | 3.4 | 0.090 |
| 46 | Alpha-actinin 2 | gi\|157951643 | 104479 | 3.4 | 0.090 |
| 61 | Collagen alpha-1 chain | gi\|293344916 | 111141 | 3.9 | 0.261 |
| 72 | Myomesin-1 (skelemin) | gi\|149036289 | 150969 | 4.6 | 0.316 |
| 72 | Myomesin-2 | gi\|281306803 | 166527 | 4.6 | 0.316 |
| 76 | Myomesin-1 (skelemin) | gi\|149036289 | 150969 | 4.8 | 0.113 |
| 76 | Munc13-3 | gi\|1763306 | 251868 | 4.8 | 0.113 |
| 81 | rCG23609, isoform CRA_b | gi\|149063939 | 217237 | 3.3 | 0.125 |
| 81 | myosin-7 isoform X1 | gi\|564386123 | 223756 | 3.3 | 0.125 |
| 81 | Ceruloplasmin | gi\|2506226 | 121758 | 3.3 | 0.125 |
| 114 | myomesin-2 | gi\|281306803 | 166527 | 3.0 | 0.320 |
| 114 | myomesin 1 (skelemin) 185kDa | gi\|149036289 | 150969 | 3.0 | 0.320 |
| 114 | AHNAK-related protein | gi\|17981389 | 10348 | 3.0 | 0.320 |
| 305 | alpha-actinin-2 | gi\|157951643 | 104479 | 6.7 | 0.325 |
| 330 | serum albumin precursor | gi\|158138568 | 71200 | 3.9 | 0.491 |
| 330 | gelsolin precursor | gi\|51854227 | 86511 | 3.9 | 0.491 |
| 330 | lon protease homolog, mitochondrial precursor | gi\|19173766 | 106437 | 3.9 | 0.491 |
| 330 | alpha-actinin-2 | gi\|157951643 | 104479 | 3.9 | 0.491 |
| 330 | E3 ubiquitin-protein ligase RNF181 | gi\|56090373 | 19773 | 3.9 | 0.491 |
| 402 | elongation factor 2 | gi\|8393296 | 96431 | 3.2 | 0.127 |
| 428 | rCG43497, isoform CRA_a | gi\|149069299 | 69277 | 4.7 | 0.013 |
| 443 | Inter-alpha-inhibitor H4 heavy chain | gi\|2292988 | 103969 | 3.1 | 0.134 |
| 469 | myosin-7 | gi\|8393807 | 223939 | 3.2 | 0.537 |
| 483 | Inter-alpha-inhibitor H4 heavy chain | gi\|2292988 | 103969 | 4.5 | 0.491 |
| 483 | KIAA0170 protein | gi\|46237657 | 68143 | 4.5 | 0.491 |
| 513 | rat testicular antigenic protein 2 | gi\|114145644 | 40828 | 3.8 | 0.101 |
| 527 | similar to RIKEN cDNA 1300017J02, isoform CRA_c [ | gi\|149018745 | 47815 | 3.7 | 0.001 |
| 527 | uncharacterized protein LOC315963 | gi\|114145796 | 52291 | 3.7 | 0.001 |
| 588 | Stress-70 protein, mitochondrial | gi\|116242506 | 74167 | 3.3 | 0.222 |
| 610 | dnaK-type molecular chaperone hsp72-ps1 - rat | gi\|347019 | 71168 | 1.7 | 0.004 |
| 610 | alpha-actinin-2 | gi\|157951643 | 104479 | 1.7 | 0.004 |
| 610 | heat shock protein 70 | gi\|415898 | 50516 | 1.7 | 0.004 |
| 637 | heat shock protein 70 | gi\|294568 | 70475 | 3.9 | 0.162 |
| 637 | dnaK-type molecular chaperone hsp72-ps1 | gi\|347019 | 71168 | 3.9 | 0.162 |
| 646 | heat shock 70kD protein 1B | gi\|47059179 | 70497 | 4.1 | 0.319 |
| 787 | dihydropyrimidinase-related protein 2 | gi\|40254595 | 62736 | 1.7 | 0.002 |
| 787 | Electron transfer flavoprotein-ubiquinone oxidoreductase, mitochondrial | gi\|52000614 | 69220 | 1.7 | 0.002 |
| 889 | vitamin D-binding protein precursor | gi\|203941 | 55471 | 3.7 | 0.077 |
| 902 | dnaK-type molecular chaperone hsp72-ps1 | gi\|347019 | 71168 | 4.6 | 0.350 |
| 902 | transitional endoplasmic reticulum ATPase | gi\|17865351 | 90145 | 4.6 | 0.350 |
| 954 | calsequestrin | gi\|988307 | 47909 | 4.1 | 0.511 |
| 954 | serine/threonine-protein phosphatase 2A 65 kDa regulatory subunit A alpha isoform | gi\|8394027 | 66275 | 4.1 | 0.511 |
| 1060 | albumin, isoform CRA_a | gi\|149033753 | 53439 | 4.0 | 0.179 |
| 1060 | fibrinogen alpha chain isoform 1 precursor | gi\|56797757 | 87526 | 4.0 | 0.179 |
| 1060 | ATP-citrate synthase | gi\|113116 | 121695 | 4.0 | 0.179 |
| 1140 | ATP synthase beta subunit | gi\|1374715 | 51171 | 3.3 | 0.503 |
| 1191 | Serine protease inhibitor | gi\|32563565 | 68536 | 5.5 | 0.389 |
| 1191 | rCG43497, isoform CRA_a | gi\|149069299 | 69277 | 5.5 | 0.389 |
| 1203 | actin, aortic smooth muscle | gi\|4501883 | 42479 | 2.1 | 0.000 |
| 1221 | citrate synthase, mitochondrial precursor | gi\|18543177 | 52260 | 6.0 | 0.099 |
| 1221 | long-chain specific acyl-CoA dehydrogenase, mitochondrial precursor | gi\|6978431 | 48340 | 6.0 | 0.099 |
| 1221 | succinyl-CoA ligase [ADP-forming] subunit beta, mitochondrial | gi\|158749584 | 50700 | 6.0 | 0.099 |
| 1244 | actin, aortic smooth muscle | gi\|4501883 | 42479 | 8.0 | 0.145 |
| 1244 | ATP synthase subunit beta, mitochondrial precursor | gi\|54792127 | 56318 | 8.0 | 0.145 |
| 1258 | creatine kinase M-type | gi\|6978661 | 43276 | 3.6 | 0.059 |
| 1258 | pyruvate dehydrogenase E1 alpha form 1 subunit | gi\|57657 | 44021 | 3.6 | 0.059 |
| 1258 | citrate synthase | gi\|89574067 | 26182 | 3.6 | 0.059 |
| 1258 | long-chain specific acyl-CoA dehydrogenase, mitochondrial precursor | gi\|6978431 | 48340 | 3.6 | 0.059 |
| 1293 | citrate synthase, mitochondrial precursor | gi\|18543177 | 52260 | 3.8 | 0.022 |
| 1293 | tubulin alpha | gi\|223556 | 51062 | 3.8 | 0.022 |
| 1309 | actin, aortic smooth muscle | gi\|4501883 | 42479 | 3.8 | 0.009 |
| 1331 | [Protein ADP-ribosylarginine] hydrolase-like protein 1 | gi\|61556810 | 40491 | 3.6 | 0.148 |
| 1331 | importin subunit alpha-6 | gi\|68341941 | - | 3.6 | 0.148 |
| 1399 | rCG41181 | gi\|149029501 | 12465 | 2.1 | 0.027 |
| 1399 | ADP-ribose pyrophosphatase, mitochondrial isoform X1 | gi\|564382772 | 33705 | 2.1 | 0.027 |
| 1399 | zinc finger protein 516 | gi\|300794570 | 126417 | 2.1 | 0.027 |
| 1432 | tubulin alpha-1B chain | gi\|34740335 | 50972 | 1.7 | 0.041 |
| 1432 | la-related protein 1 isoform X2 | gi\|672067583 | 123313 | 1.7 | 0.041 |
| 1490 | sarcolemmal membrane-associated protein isoform X9 | gi\|564387776 | 93149 | 3.1 | 0.260 |
| 1490 | heat shock protein (hsp60) precursor | gi\|56383 | 61141 | 3.1 | 0.260 |
| 1490 | Annexin A6 | gi\|1351943 | 76204 | 3.1 | 0.260 |
| 1490 | rCG45627, isoform CRA_c | gi\|149039438 | 217747 | 3.1 | 0.260 |
| 1500 | pyruvate dehydrogenase E1 component subunit beta, mitochondrial precursor | gi\|56090293 | 39383 | 4.3 | 0.301 |
| 1516 | creatine kinase M-type | gi\|6671762 | 43302 | 9.8 | 0.488 |
| 1516 | aldose reductase | gi\|6978491 | 36343 | 9.8 | 0.488 |
| 1516 | 60S acidic ribosomal protein P0 | gi\|11693176 | 34407 | 9.8 | 0.488 |
| 1516 | long-chain specific acyl-CoA dehydrogenase, mitochondrial precursor | gi\|6978431 | 48340 | 9.8 | 0.488 |
| 1516 | malate dehydrogenase, cytoplasmic | gi\|15100179 | 36673 | 9.8 | 0.488 |
| 1516 | annexin II | gi\|9247200 | 39306 | 9.8 | 0.488 |
| 1564 | adiponectin precursor | gi\|21426809 | 26535 | 2.1 | 0.030 |
| 1564 | Leukotriene A-4 hydrolase | gi\|266480 | 69914 | 2.1 | 0.030 |
| 1584 | Tropomyosin alpha-3 chain | gi\|148840439 | 29273 | 2.7 | 0.013 |
| 1603 | actin, gamma 2, isoform CRA_b | gi\|149036532 | 34004 | 4.5 | 0.101 |
| 1603 | brain creatine kinase | gi\|203470 | 43040 | 4.5 | 0.101 |
| 1603 | PDZ domain containing 3, isoform CRA_b | gi\|149027290 | 295650 | 4.5 | 0.101 |
| 1604 | actin, gamma 2, isoform CRA_b | gi\|149036532 | 34004 | 2.4 | 0.005 |
| 1604 | lipocortin V | gi\|2981437 | 33944 | 2.4 | 0.005 |
| 1641 | actin, alpha skeletal muscle | gi\|4501881 | 42450 | 2.8 | 0.028 |
| 1685 | L-lactate dehydrogenase B chain | gi\|6981146 | 36944 | 2.8 | 0.009 |
| 1691 | actin, aortic smooth muscle | gi\|4501883 | 42479 | 3.1 | 0.017 |
| 1691 | actin, cytoplasmic 1 | gi\|4501885 | 42136 | 3.1 | 0.017 |
| 1699 | purine nucleoside phosphorylase isoform X1 | gi\|564385910 | 32636 | 4.2 | 0.300 |
| 1699 | 3-hydroxyisobutyrate dehydrogenase, partial | gi\|556389 | 36839 | 4.2 | 0.300 |
| 1774 | 14-3-3 protein gamma | gi\|9507245 | 28498 | 6.9 | 0.411 |
| 1774 | uncharacterized protein LOC499135 | gi\|67846090 | 79383 | 6.9 | 0.411 |
| 1780 | tyrosine-protein phosphatase non-receptor type substrate 1-like isoform X1 | gi\|672016706 | 30260 | 4.4 | 0.104 |
| 1781 | actin, aortic smooth muscle | gi\|4501883 | 42479 | 7.0 | 0.386 |
| 1781 | ATP synthase subunit gamma, mitochondrial | gi\|728931 | 30243 | 7.0 | 0.386 |
| 1807 | 14-3-3 zeta isoform | gi\|1051270 | 27997 | 5.9 | 0.288 |
| 1840 | Heat shock protein beta-1; Short=HspB1 | gi\|1170367 | 22950 | 3.3 | 0.567 |
| 1941 | myosin light chain 4 | gi\|157823723 | 21383 | 4.7 | 0.068 |
| 2004 | peroxiredoxin-2 | gi\|8394432 | 21983 | 2.2 | 0.021 |
| 2011 | peroxiredoxin-2 | gi\|8394432 | 21983 | 2.3 | 0.024 |
| 2035 | actin, gamma 2, isoform CRA_b | gi\|149036532 | 34004 | 8.6 | 0.382 |
| 2062 | myosin regulatory light chain 2, atrial isoform isoform X1 | gi\|564384290 | 19681 | 3.2 | 0.014 |
| 2147 | histone H2B type 1-C/E/F/G/I | gi\|27693390 | 13898 | 4.5 | 0.436 |
| 2147 | Methylmalonate-semialdehyde dehydrogenase [acylating], mitochondrial | gi\|400269 | 58339 | 4.5 | 0.436 |
| 2147 | histone H4 | gi\|672051605 | 11360 | 4.5 | 0.436 |
| 2147 | uncharacterized protein LOC100910366 | gi\|672030073 | 47515 | 4.5 | 0.436 |
| 2147 | rCG38845, isoform CRA_a | gi\|149035966 | 13078 | 4.5 | 0.436 |
| 2165 | myosin regulatory light chain 2, atrial isoform isoform X1 | gi\|564384290 | 19681 | 3.8 | 0.170 |
| 2189 | myosin light chain | gi\|205474 | 20977 | 4.0 | 0.374 |
| 2194 | alpha-myosin heavy chain, partial | gi\|554475 | 19239 | 6.2 | 0.450 |
| 2208 | actin, gamma 2, isoform CRA_b | gi\|149036532 | 34004 | 3.4 | 0.306 |
| 2208 | major beta-hemoglobin | gi\|204570 | 16125 | 7.1 | 0.212 |
| 2254 | rCG32620, isoform CRA_d | gi\|149054505 | 14874 | 3.6 | 0.108 |
| 2279 | histidine triad nucleotide-binding protein 1 | gi\|33468857 | 13910 | 4.3 | 0.065 |
| 151 | vinculin (predicted), isoform CRA_a | gi\|149031250 | 124266 | -4.0 | 0.564 |
| 151 | pyruvate carboxylase, mitochondrial isoform X2 | gi\|564332044 | 141337 | -4.0 | 0.564 |
| 367 | heat shock protein HSP 90-beta | gi\|40556608 | 83655 | -1.7 | 0.037 |
| 367 | heat shock protein HSP 90-alpha | gi\|28467005 | 85259 | -1.7 | 0.037 |
| 517 | radixin | gi\|40804379 | 68742 | -7.8 | 0.025 |
| 517 | moesin | gi\|13540689 | 67910 | -7.8 | 0.025 |
| 592 | alpha-1 major acute phase protein prepeptide, partial | gi\|205308 | 48576 | -3.1 | 0.334 |
| 634 | LMW T-kininogen I precursor | gi\|205085 | 49023 | -5.4 | 0.312 |
| 634 | rCG46767, isoform CRA_c | gi\|149064296 | 66930 | -5.4 | 0.312 |
| 634 | Annexin A6 | gi\|1351943 | 76204 | -5.4 | 0.312 |
| 634 | actin, alpha 1, skeletal muscle, isoform CRA_a | gi\|149043182 | 52115 | -5.4 | 0.312 |
| 645 | Myristoylated alanine-rich C-kinase substrate | gi\|266495 | 29848 | -4.7 | 0.198 |
| 712 | Optineurin | gi\|62286963 | 67824 | -2.1 | 0.046 |
| 712 | LMW T-kininogen I precursor | gi\|205085 | 49023 | -2.1 | 0.046 |
| 718 | Annexin A6 | gi\|1351943 | 76204 | -2.9 | 0.028 |
| 718 | EH domain-containing protein 2 | gi\|67846074 | 61412 | -2.9 | 0.028 |
| 718 | dnaK-type molecular chaperone hsp72-ps1 | gi\|347019 | 71168 | -2.9 | 0.028 |
| 819 | heat shock protein (hsp60) precursor | gi\|56383 | 61141 | -2.2 | 0.003 |
| 819 | antithrombin-III precursor | gi\|58865630 | 52840 | -2.2 | 0.003 |
| 819 | Alpha-2-HS-glycoprotein | gi\|231468 | 38953 | -2.2 | 0.003 |
| 819 | alpha-actinin-2 | gi\|157951643 | 104479 | -2.2 | 0.003 |
| 828 | heat shock protein (hsp60) precursor | gi\|56383 | 61141 | -2.2 | 0.033 |
| 828 | tubulin alpha | gi\|223556 | 51062 | -2.2 | 0.033 |
| 828 | T-complex protein 1 subunit theta | gi\|347800699 | 60261 | -2.2 | 0.033 |
| 857 | nucleosome assembly protein 1-like 4 | gi\|58865912 | 44174 | -1.8 | 0.029 |
| 857 | rCG50690, partial | gi\|149031970 | 21249 | -1.8 | 0.029 |
| 871 | UV excision repair protein RAD23 homolog B | gi\|70778952 | 43541 | -2.6 | 0.003 |
| 878 | heat shock protein (hsp60) precursor | gi\|56383 | 61141 | -2.8 | 0.008 |
| 878 | tubulin alpha-1B chain | gi\|34740335 | 50972 | -2.8 | 0.008 |
| 878 | heterogeneous nuclear ribonucleoprotein K isoform 2 | gi\|13384620 | 51300 | -2.8 | 0.008 |
| 895 | tubulin beta-5 chain | gi\|7106439 | 50207 | -3.3 | 0.215 |
| 900 | iodothyronine 5' monodeiodinase, partial | gi\|202549 | 54459 | -3.3 | 0.028 |
| 945 | desmin | gi\|11968118 | 53495 | -5.1 | 0.034 |
| 945 | tubulin alpha-1B chain | gi\|34740335 | 50972 | -5.1 | 0.034 |
| 945 | cardiac calsequestrin | gi\|2150120 | 42135 | -5.1 | 0.034 |
| 946 | vitamin D-binding protein precursor | gi\|203941 | 55471 | -3.4 | 0.271 |
| 946 | tubulin alpha-1B chain | gi\|34740335 | 50972 | -3.4 | 0.271 |
| 946 | glucose-6-phosphate isomerase | gi\|46485440 | 63000 | -3.4 | 0.271 |
| 950 | group specific component, isoform CRA_a | gi\|149033741 | 41612 | -3.2 | 0.145 |
| 950 | glucose-6-phosphate isomerase | gi\|46485440 | 63000 | -3.2 | 0.145 |
| 1041 | Chain A, Rat Liver F1-Atpase | gi\|6729934 | 55389 | -3.6 | 0.031 |
| 1041 | albumin, isoform CRA_a | gi\|149033753 | 53439 | -3.6 | 0.031 |
| 1041 | Pdhx protein | gi\|60688224 | 41078 | -3.6 | 0.031 |
| 1041 | aldehyde dehydrogenase, mitochondrial precursor | gi\|14192933 | 57092 | -3.6 | 0.031 |
| 1072 | serum albumin precursor | gi\|158138568 | 71200 | -3.2 | 0.203 |
| 1072 | ATP synthase, mitochondrial F1 complex, alpha subunit | gi\|149029483 | 54660 | -3.2 | 0.203 |
| 1076 | cytochrome b-c1 complex subunit 1, mitochondrial precursor | gi\|51948476 | 53668 | -4.2 | 0.201 |
| 1076 | brain creatine kinase | gi\|203470 | 43040 | -4.2 | 0.201 |
| 1076 | actin, alpha skeletal muscle | gi\|4501881 | 42450 | -4.2 | 0.201 |
| 1128 | brain creatine kinase | gi\|203470 | 43040 | -3.9 | 0.273 |
| 1189 | 40S ribosomal protein SA | gi\|8393693 | 32946 | -6.0 | 0.034 |
| 1220 | long-chain specific acyl-CoA dehydrogenase, mitochondrial precursor | gi\|6978431 | 48340 | -3.0 | 0.151 |
| 1220 | citrate synthase, mitochondrial precursor | gi\|18543177 | 52260 | -3.0 | 0.151 |
| 1220 | troponin T | gi\|203656 | 35679 | -3.0 | 0.151 |
| 1220 | brain creatine kinase | gi\|203470 | 43040 | -3.0 | 0.151 |
| 1226 | long-chain specific acyl-CoA dehydrogenase, mitochondrial precursor | gi\|6978431 | 48340 | -4.0 | 0.398 |
| 1226 | Suclg2 protein | gi\|59808474 | 42050 | -4.0 | 0.398 |
| 1226 | citrate synthase | gi\|89574067 | 26182 | -4.0 | 0.398 |
| 1235 | long-chain specific acyl-CoA dehydrogenase, mitochondrial precursor | gi\|6978431 | 48340 | -5.5 | 0.279 |
| 1235 | citrate synthase, mitochondrial precursor | gi\|18543177 | 52260 | -5.5 | 0.279 |
| 1235 | cytochrome b-c1 complex subunit 2, mitochondrial precursor | gi\|55741544 | 48437 | -5.5 | 0.279 |
| 1235 | troponin T | gi\|203656 | 35679 | -5.5 | 0.279 |
| 1235 | heat shock protein 90 | gi\|256089 | 83690 | -5.5 | 0.279 |
| 1239 | NDRG1 related protein NDRG2a2 | gi\|17977872 | 39672 | -6.7 | 0.298 |
| 1319 | Chain A, Crystal Structure Of Rat Short Chain Acyl-Coa Dehydrogenase | gi\|20150494 | 42502 | -3.5 | 0.203 |
| 1319 | creatine kinase M-type | gi\|6978661 | 43276 | -3.5 | 0.203 |
| 1319 | short/branched chain specific acyl-CoA dehydrogenase, mitochondrial | gi\|6978433 | 48362 | -3.5 | 0.203 |
| 1383 | histone H2B type 1 | gi\|12025526 | 13982 | -3.1 | 0.069 |
| 1383 | nucleolar protein 12 | gi\|60678272 | 25170 | -3.1 | 0.069 |
| 1383 | similar to IL25 | gi\|149063935 | 19208 | -3.1 | 0.069 |
| 1383 | Ba1-647 | gi\|33086640 | 43229 | -3.1 | 0.069 |
| 1421 | haptoglobin precursor | gi\|60097941 | 39178 | -3.1 | 0.121 |
| 1421 | L-lactate dehydrogenase B chain | gi\|6981146 | 36944 | -3.1 | 0.121 |
| 1430 | Ba1-647 | gi\|33086640 | 43229 | -4.7 | 0.448 |
| 1430 | cardiac myosin heavy chain 21/26, partial | gi\|205576 | 49631 | -4.7 | 0.448 |
| 1430 | aldehyde reductase AFAR2 subunit | gi\|22652804 | 41232 | -4.7 | 0.448 |
| 1431 | Ba1-647 | gi\|33086640 | 43229 | -5.7 | 0.203 |
| 1431 | monocarboxylate transporter 1 | gi\|6981542 | - | -5.7 | 0.203 |
| 1485 | N(G),N(G)-dimethylarginine dimethylaminohydrolase 1 | gi\|11560131 | 31903 | -5.4 | 0.354 |
| 1485 | malate dehydrogenase, cytoplasmic | gi\|15100179 | 36673 | -5.4 | 0.354 |
| 1485 | L-lactate dehydrogenase B chain | gi\|6981146 | 36944 | -5.4 | 0.354 |
| 1485 | aldose reductase | gi\|6978491 | 36343 | -5.4 | 0.354 |
| 1485 | annexin II | gi\|9247200 | 39306 | -5.4 | 0.354 |
| 1485 | isocitrate dehydrogenase [NAD] subunit alpha, mitochondrial precursor | gi\|16758446 | 40156 | -5.4 | 0.354 |
| 1489 | lipocortin I | gi\|235879 | 39221 | -3.3 | 0.050 |
| 1489 | PDZ and LIM domain protein 1 | gi\|8393153 | 36071 | -3.3 | 0.050 |
| 1489 | aldose reductase | gi\|6978491 | 36343 | -3.3 | 0.050 |
| 1495 | desmoplakin | gi\|109504778 | 335170 | -5.0 | 0.068 |
| 1501 | Ba1-647 | gi\|33086640 | 43229 | -3.4 | 0.044 |
| 1501 | L-lactate dehydrogenase B chain | gi\|6981146 | 36944 | -3.4 | 0.044 |
| 1501 | malate dehydrogenase, cytoplasmic | gi\|15100179 | 36673 | -3.4 | 0.044 |
| 1501 | annexin II | gi\|9247200 | 39306 | -3.4 | 0.044 |
| 1591 | 14-3-3 protein epsilon | gi\|13928824 | 29316 | -3.3 | 0.453 |
| 1624 | 14-3-3 protein epsilon | gi\|13928824 | 29316 | -4.6 | 0.482 |
| 1639 | troponin T | gi\|203656 | 35679 | -8.9 | 0.391 |
| 1639 | Clusterin | gi\|461756 | 52124 | -8.9 | 0.391 |
| 1730 | 14-3-3 zeta isoform | gi\|1051270 | 27997 | -10.2 | 0.011 |
| 1730 | ADP/ATP translocase 1 | gi\|32189355 | 33252 | -10.2 | 0.011 |
| 1730 | phosphoglycerate mutase 2 | gi\|8393948 | 28950 | -10.2 | 0.011 |
| 1790 | Chain A, 2-Enoyl-Coa Hydratase | gi\|2392291 | 28625 | -3.4 | 0.471 |
| 1790 | triosephosphate isomerase | gi\|538426 | 27543 | -3.4 | 0.471 |
| 1790 | pyridoxine-5'-phosphate oxidase | gi\|12018270 | 30591 | -3.4 | 0.471 |
| 1790 | ADP/ATP translocase 1 | gi\|32189355 | 33252 | -3.4 | 0.471 |
| 1806 | troponin I, cardiac muscle | gi\|8394469 | 24287 | -3.3 | 0.012 |
| 1806 | Tpi1 protein, partial | gi\|38512111 | 27340 | -3.3 | 0.012 |
| 1806 | dynactin subunit 2 | gi\|51948450 | 44263 | -3.3 | 0.012 |
| 1857 | preproapolipoprotein A-I | gi\|55747 | 30140 | -5.1 | 0.306 |
| 1857 | Ig kappa chain C region, A allele | gi\|125142 | 11938 | -5.1 | 0.306 |
| 1857 | Heat shock protein beta-1 | gi\|1170367 | 22950 | -5.1 | 0.306 |
| 1881 | ATP synthase F(0) complex subunit B1, mitochondrial precursor | gi\|19705465 | 28993 | -6.9 | 0.052 |
| 1881 | peroxiredoxin 3 | gi\|149040547 | 28637 | -6.9 | 0.052 |
| 1881 | Ig kappa chain precursor | gi\|92401 | 26807 | -6.9 | 0.052 |
| 1881 | dihydrolipoamide S-acetyltransferase | gi\|2117706 | 48160 | -6.9 | 0.052 |
| 2015 | protein deglycase DJ-1 isoform 2 | gi\|16924002 | 20246 | -6.6 | 0.019 |
| 2015 | Thioredoxin-dependent peroxide reductase, mitochondrial | gi\|118597399 | 28633 | -6.6 | 0.019 |
| 2015 | glutathione peroxidase | gi\|9247190 | 22594 | -6.6 | 0.019 |
| 2044 | transcription factor BTF3 homolog 4 | gi\|29789195 | 17260 | -3.0 | 0.361 |
| 2067 | nucleolar protein 12 | gi\|60678272 | 25170 | -3.3 | 0.476 |
| 2074 | septin-8 isoform X1 | gi\|564371739 | 58414 | -5.7 | 0.296 |
| 2091 | 5-demethoxyubiquinone hydroxylase, mitochondrial | gi\|472235294 | 24139 | -1.8 | 0.006 |
| 2091 | thiamine transporter 1 | gi\|71795619 | 56485 | -1.8 | 0.006 |
| 2112 | myosin regulatory light chain 2, atrial isoform | gi\|157821295 | 16933 | -3.5 | 0.024 |
| 2112 | olfactory receptor Olr46 | gi\|47577455 | 36156 | -3.5 | 0.024 |
| 2183 | myoglobin | gi\|11024650 | 17217 | -9.6 | 0.030 |
| 2183 | cytochrome c oxidase subunit 4 isoform 1, mitochondrial precursor | gi\|8393180 | 19573 | -9.6 | 0.030 |
| 2193 | myoglobin | gi\|11024650 | 17217 | -4.0 | 0.004 |
| 2195 | myoglobin | gi\|11024650 | 17217 | -3.7 | 0.004 |
| 2197 | myoglobin | gi\|11024650 | 17217 | -3.5 | 0.051 |
| 2212 | prealbumin | gi\|205982 | 15880 | -6.8 | 0.308 |
