# Supplementary material for: Identification of dysregulation of atrial proteins in rats with chronic obstructive apnea using two‐dimensional polyacrylamide gel electrophoresis and mass spectrometry
Source: J Cell Mol Med. 2019 Feb 12;23(4):3016–20. doi: 10.1111/jcmm.14131 (PMC6433690; doi:10.1111/jcmm.14131)
Supplement: Supplementary file 9 [file JCMM-23-3016-s009.docx]

| **Spot #** | **Protein** | **NCBInr Accession no** | **Mr (kDa)** | **Fold Change** | **P value** |
| --- | --- | --- | --- | --- | --- |
| 72 | Myomesin-1 (skelemin) | gi\|149036289 | 150969 | 4.75074627 | 0.202666 |
| 72 | Myomesin-2 | gi\|281306803 | 166527 | 4.75074627 | 0.202666 |
| 330 | serum albumin precursor | gi\|158138568 | 71200 | 10.5672018 | 0.412173 |
| 330 | gelsolin precursor | gi\|51854227 | 86511 | 10.5672018 | 0.412173 |
| 330 | lon protease homolog, mitochondrial precursor | gi\|19173766 | 106437 | 10.5672018 | 0.412173 |
| 330 | alpha-actinin-2 [Mus musculus] | gi\|157951643 | 104479 | 10.5672018 | 0.412173 |
| 330 | E3 ubiquitin-protein ligase RNF181 | gi\|56090373 | 19773 | 10.5672018 | 0.412173 |
